# MFF-AE: Enhanced Quality Control for Proteomics Mass Spectrometry Data via Multi-Scale Feature Fusion

**DOI:** 10.3390/ijms27052121

**Published:** 2026-02-25

**Authors:** Guangkui Fan, Xinyu Ji, Hunyue Liao, Bo Meng, Duotao Pan, Jinze Huang, Yang Zhao

**Affiliations:** 1 College of Information, Shenyang University of Chemical Technology, Shenyang 110142, China; fan694558432@126.com (G.F.);; 2 Technology Innovation Center of Mass Spectrometry for State Market Regulation, Center for Advanced Measurement Science, National Institute of Metrology, Beijing 100029, China; jixy@nim.ac.cn (X.J.); liaohy@nim.ac.cn (H.L.); mengbo@nim.ac.cn (B.M.)

**Keywords:** quality control, proteomics, deep learning, feature fusion, autoencoder

## Abstract

Mass spectrometry (MS) is a core analytical tool in proteomics, and the quality of the generated data directly determines the effectiveness of downstream analyses and the reliability of final research conclusions. While MS is also widely used in other omics applications, this study focuses on label-free quantitative proteomics, where samples are represented as protein-abundance matrices derived from MaxQuant. However, MS data are typically characterized by high dimensionality and substantial noise, posing serious challenges for quality control (QC). Existing QC methods have limited feature extraction capabilities and struggled to capture the key information embedded in the data, resulting in poor performance in identifying anomalous samples. Here, we propose the Multi-Scale Feature Fusion-based Autoencoder (MFF-AE). This deep learning-based anomaly detection model achieves precise identification of anomalous samples by integrating both global and local data features. The model consists of three modules: an autoencoder-based backbone network that efficiently embeds raw data into a low-dimensional semantic space, a local feature extraction and fusion module designed to capture and integrate multi-scale features within MS data, and a sample identification module that enhances discriminative representations to enable accurate anomaly detection. To evaluate the effectiveness of the proposed model, we conduct extensive experiments on a benchmark dataset with synthesized anomalies. Quantitative results on the benchmark dataset show that, compared with 15 baseline models from statistical learning, deep learning, and ensemble learning, our model consistently achieves the best performance across key metrics. Furthermore, through linear relationship analysis on real-world clinical datasets, the exclusion of outlier samples significantly increased the statistical significance and fold change in the identified differential proteins. Overall, the proposed model establishes a solid data foundation, paving the way for downstream mechanistic studies and target discovery.

## 1. Introduction

Proteomics provides an essential foundation for elucidating disease mechanisms and discovering biomarkers and therapeutic targets, and mass spectrometry (MS), with its high throughput and high sensitivity, has become a core analytical technology in proteomics research [1,2]. However, MS data are often characterized by high dimensionality, high noise levels, and distributional heterogeneity, making quality control (QC) a critical step for ensuring the reliability of downstream analyses. Large-scale proteomic data inevitably suffer from system noise, batch effects, and cross-experiment variation; these issues contribute to data inconsistency and false-positive findings [3,4]. Unlike other large-scale data, technical anomalies in MS data typically manifest in localized and subtle forms. Although their individual effects may appear minor, they can accumulate in the high-dimensional feature space and compromise differential analysis and biomarker identification [5]. In addition, the high-throughput nature of multi-step MS experiments further amplifies these subtle deviations, further complicating anomaly detection.

Facing such localized anomalies, existing QC approaches either focus excessively on global distributional characteristics or fail to maintain discriminative capability in high-dimensional sparse data. Traditional statistical models, such as Gaussian Mixture Models (GMM), rely on specific distributional assumptions (e.g., Gaussian), but protein expression profiles exhibit non-Gaussian and multimodal characteristics, causing such assumption-dependent methods to often fail. Proximity-based methods, such as K-nearest neighbors (KNN) and Local Outlier Factor (LOF), detect anomalies by estimating local density or distance relationships among samples [6,7,8]. Although these methods improve sensitivity to local anomalies, they suffer from the ‘curse of dimensionality’. In feature spaces with thousands of dimensions, distance measures lose discriminability, rendering pairwise sample distances nearly indistinguishable and undermining detection performance [9]. Ensemble methods such as Isolation Forest, Deep Isolation Forest (DIF), and FeatureBagging enhance model stability through randomization strategies [10,11]. However, they struggle to model the high-order nonlinear associations in proteomic data and exhibit limited generalization under typical small-sample conditions [12,13].

With the rise of deep learning, unsupervised autoencoder (AE)-based methods have offered new possibilities for anomaly detection [14]. AEs measure sample deviation from normal patterns via reconstruction error and can preserve overall data structure. Variational autoencoders (VAE) further improve generalization by incorporating probabilistic modeling and learning a continuous, regularized latent space [15]. Other deep methods, such as DeepSVDD, optimize the latent space geometrically to directly separate normal and anomalous samples [16]. However, these methods tend to focus on global distributional structure and overlook the fine-grained local patterns where technical anomalies typically arise in proteomics applications [17]. Furthermore, unsupervised models are susceptible to overfitting on anomalous samples during training, thereby diminishing the diagnostic utility of reconstruction error for anomaly detection [18,19].

Multi-scale feature fusion has been shown to significantly enhance sensitivity to local perturbations in image, sequence, and spectral data, providing inspiration for addressing localized and subtle anomalies in proteomics [20,21]. Drawing on this insight, we propose a novel QC method for MS data, termed the Multi-scale Feature Fusion Autoencoder (MFF-AE). The core idea is to jointly model both the global distributional patterns and the localized subtle anomalies of the data within an autoencoder framework. To achieve this, we design two key modules within the autoencoder. First, the Multi-Scale Fusion (MSF) module partitions shallow encoder outputs into multiple subspaces to extract local patterns, subsequently fusing them with global features. Second, we introduce a Class-Specific Identification (CLS) module for self-supervised instance discrimination on the global representation $F_{global}$. The CLS loss is used only during training to regularize representation learning, while inference relies on reconstruction-based anomaly scoring. The method can be directly applied to protein quantification matrices generated by MaxQuant (v2.0.3.0). Experiments on the benchmark dataset compared our method with 15 representative baselines spanning statistical, deep, and ensemble learning. Results show that our method exhibits dominant performance in key metrics on the area under the receiver operating characteristic (ROC) curve (AUC-ROC) and the area under the precision-recall(PR) curve (AUC-PR). And on the real-world clinical datasets, differential expression analysis performed after MFF-AE QC yields more stable statistical significance and fold changes, indicating that the model effectively identifies anomalous samples while improving the reliability of downstream analyses without introducing systematic bias. This provides a more trustworthy data foundation for mechanistic studies and biomarker discovery.

## 2. Results

### 2.1. Performance Evaluation

To comprehensively evaluate model performance, we selected 15 representative anomaly detection algorithms as baseline models for comparison. These baselines cover traditional statistical learning methods, mainstream deep learning approaches, and classical ensemble methods, ensuring a thorough and balanced comparison. All baseline models were implemented using PyOD [22] or their official implementations, with default hyperparameters applied to maintain reproducibility.

**Figure 1:**
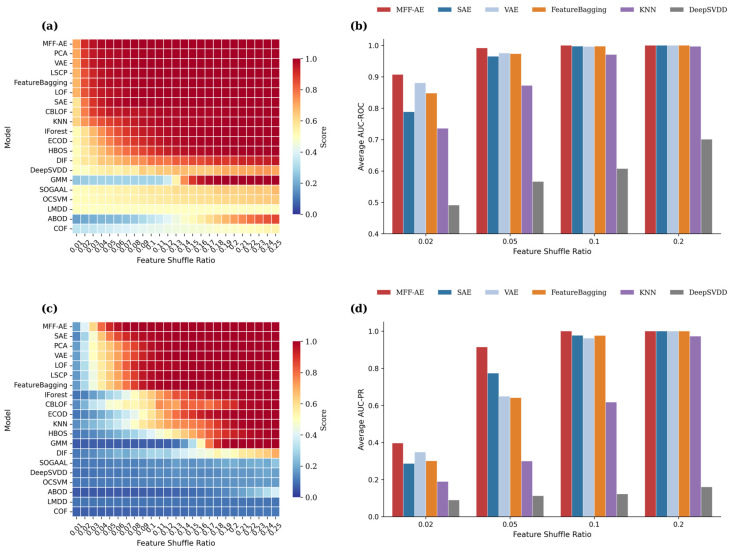
Performance comparison of MFF-AE with other baseline models under different feature shuffle ratios. (**a**,**c**) present the performance heatmaps for the area under the receiver operating characteristic (ROC) curve (AUC-ROC) and the area under the precision-recall (PR) curve (AUC-PR), respectively (ratio range: 0.01–0.25). Models are sorted by mean performance in descending order; MFF-AE (top row) exhibits rapid convergence and global stability at low perturbation levels. Each heatmap entry corresponds to the mean sample-level area under the curve (AUC) value computed against the simulation ground-truth labels under the given shuffle ratio, averaged over ten independent trials. (**b**,**d**) display the grouped bar charts for AUC-ROC and AUC-PR at key perturbation snapshots (0.02, 0.05, 0.10, 0.20). These charts quantify the specific performance differences between MFF-AE (red bars) and representative deep learning baselines (e.g., standard autoencoder (SAE), variational autoencoders (VAE), DeepSVDD) as well as traditional models (e.g., FeatureBagging, k-nearest neighbors (KNN)). Each data point represents the average of ten independent trials.

We adopted AUC-ROC and AUC-PR metrics to assess performance stability. As illustrated in the AUC-ROC heatmap Figure 1a, MFF-AE (top row) maintains a consistent deep-red coloration across the perturbation range of 0.01 to 0.25, indicating that it achieves performance saturation even at low perturbation levels. In contrast, deep learning baselines such as DeepSVDD and SOGAAL exhibit a slower color transition, indicating a lag in performance improvement. The bar chart Figure 1b further quantifies this difference: at a feature shuffle ratio of 0.05, the AUC-ROC of MFF-AE rapidly climbs to 0.99. While DeepSVDD (0.57) and SOGAAL (0.53) show performance gains, they remain at lower levels; even strong baselines like the standard autoencoder (SAE; 0.96) and FeatureBagging (0.97) exhibit a growth rate slightly slower than that of MFF-AE. This demonstrates MFF-AE’s superior sensitivity to subtle anomaly patterns.

In the AUC-PR metric, which is critical for class-imbalanced scenarios, the difference in convergence speed is even more pronounced. The heatmap in Figure 1c shows that the top layer representing MFF-AE achieves high values early on, whereas the majority of deep models in the lower layers remain at low values during low perturbation stages, increasing only gradually as perturbation intensifies. The bar chart in Figure 1d confirms this gap: at a perturbation ratio of 0.10, MFF-AE stabilizes at a score of 1.0, while models like DeepSVDD and SOGAAL, despite showing an upward trend, remain in the 0.10–0.20 range. Additionally, while the bar height of principal component analysis (PCA) increases with higher noise conditions, its rate of improvement is visibly inferior to that of MFF-AE, suggesting that MFF-AE possesses higher efficiency in noise resistance and information preservation.

Overall, by integrating global and local features, MFF-AE demonstrates early performance saturation in the heatmaps and significant quantitative advantages in the bar charts. The results indicate that while baseline models improve as perturbation increases, MFF-AE identifies anomalies more rapidly and accurately, providing reliable support for subsequent biomarker discovery.

### 2.2. Module Parameter Optimization Results

To quantify the contribution of each component to detecting subtle anomalies, we conducted an ablation study under the most challenging low-perturbation regime (feature perturbation ratio fixed at 0.01). We compared four model variants: (1) SAE, the standard autoencoder serving as a naive baseline; (2) AE-CLS, the variant equipped with the Class-Specific Identification module but lacking multi-scale fusion; (3) AE-MSF, which incorporates the multi-scale feature fusion and reconstruction loss module; and (4) the full MFF-AE model integrating all components. The results are summarized in Table 1.

The standard AE yielded an AUC-ROC of 0.580. The addition of the classification module (AE-CLS) significantly improved this to 0.641. The introduction of the MSF module further markedly improved performance. Compared to the AE-CLS variant, AE-MSF increased AUC-ROC from 0.641 to 0.705. The complete MFF-AE model boosted AUC-ROC to 0.721 while reducing its standard deviation from 0.035 to 0.031.

A similar trend was observed for AUC-PR. While the standard AE achieved a score of 0.133, MFF-AE improved the score to 0.173 (compared to 0.141 for AE-CLS), with the standard deviation decreasing from 0.027 to 0.024. These consistent improvements across metrics demonstrate that the full model configuration not only enhances accuracy but also delivers greater stability.

The MSF module captures subtle, coordinated deviations across subsets of proteins. It is not designed to highlight single marker proteins. This setting involves weak perturbations that are distributed across features. Such signals can be diluted in a single-scale representation. MSF partitions the feature space and retains subspace-specific deviations. This mechanism is consistent with the gain from AE-CLS to AE-MSF in Table 1.

### 2.3. Individual Feature Visualization

**Figure 2:**
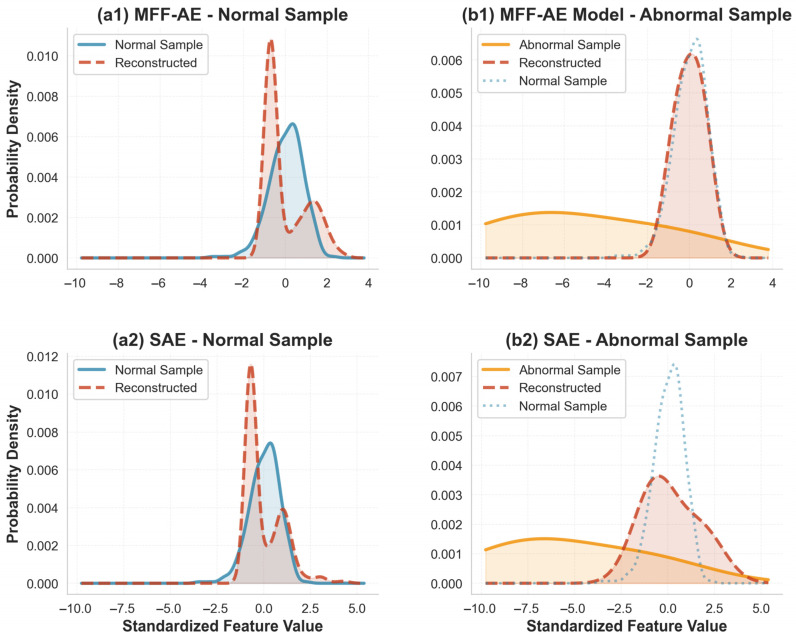
Kernel density estimation results for the O75340 protein feature in the HeLa dataset. The left column shows the distributions of normal samples before and after reconstruction, and the right column shows the corresponding distributions for anomalous samples, each compared against the distribution of normal training data. The x-axis represents the standardized feature values of the protein, and the y-axis denotes the probability density, indicating the relative likelihood of observing data points within a given unit interval. Subplots (**a1**,**b1**) correspond to the results of the proposed MFF-AE model, while subplots (**a2**,**b2**) correspond to those of the baseline SAE model. Shaded (filled) areas highlight the corresponding kernel density curves and follow the same color coding as the line plots.

Kernel Density Estimation (KDE), as a non-parametric density estimation method, provides a smooth representation of data distributions and avoids artificial discontinuities and distortion caused by binning. It enables a more intuitive understanding of the model’s reconstruction behavior [23]. Therefore, we employed KDE to visualize the distributions of samples before and after reconstruction. Taking the feature values of protein O75340 as an example, the results are shown in Figure 2. For normal samples, the reconstructed distributions from both MFF-AE and the baseline Autoencoder model closely resemble the true distribution of normal samples, with the majority of values concentrated around the sample peak (Figure 2(a1,a2)). This indicates that both models can effectively learn the structure and distribution of normal sample data. The difference is mainly observed in the reconstruction of anomalous samples. In the baseline SAE, the reconstructed distribution of anomalous samples remains closer to their original input distribution compared with MFF-AE (Figure 2(b2)). In contrast, the reconstructed distribution of MFF-AE exhibits an overall shift toward the distribution of normal samples, with only partial overlap between the reconstructed anomalies and the true anomalous distribution (Figure 2(b1)). This indicates that MFF-AE tends to pull anomalous samples toward the normal distribution during reconstruction. Consequently, anomalous samples exhibit a significantly larger reconstruction error than normal samples, making them easier to distinguish based on the magnitude of this deviation.

### 2.4. Biological Validation

To minimize potential interference from tissue-type differences in anomaly detection calling, we performed anomaly detection separately within the tumor and normal adjacent tissues groups. In the lung adenocarcinoma (LUAD) cohort, MFF-AE identified six anomalous samples (approximately 2.9%), including three tumor samples (LADC.03T, LADC.26T, and LADC.90T) and three normal adjacent tissues samples (LADC.43N, LADC.06N, and LADC.92N). Notably, the three anomalous tumor samples were fully consistent with those reported in an independent benchmark study [24], providing external support for our detection results. All six samples were excluded from subsequent differential expression analyses.

To assess effectiveness of removing anomalous samples, we examined the concordance of differential expression results before and after QC [25,26]. Among 11,286 quantified proteins, 4187 differentially expressed proteins (DEPs) were identified before QC and 4178 after QC (criteria: Benjamini-Hochberg (BH)-adjusted *p*-value < 0.01 and |log2 fold change (log2 FC)| > 2). Of these, 4111 proteins overlapped exactly between the two analyses (approximately 98.2%). This high overlap indicates that excluding anomalous samples did not materially alter the overall differential expression landscape.

**Figure 3:**
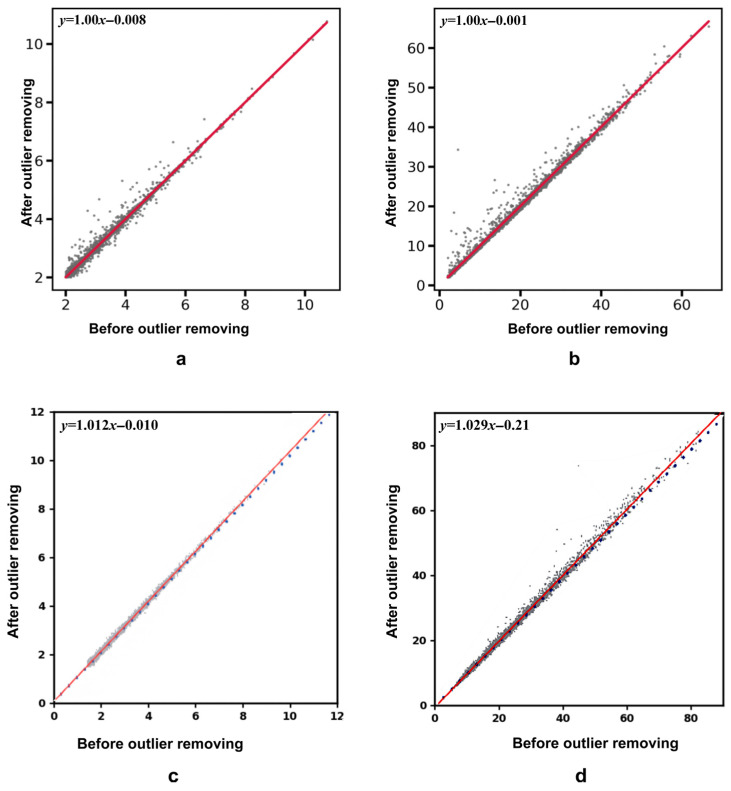
Preservation of biological signals after MFF-AE and SEAOP outlier removal. Scatter plots show the concordance of (**a**,**c**) ${log}_{2}FC$ and (**b**,**d**) −${log}_{10}$(BH-adjusted *p*-value) before vs. after QC. Panels (**a**,**b**) show MFF-AE results from this study, and panels (**c**,**d**) show SEAOP results reproduced from Ref. [24]. Differentially expressed proteins (DEPs) were defined as BH-adjusted *p*-value < 0.01 and |${log}_{2}FC$| > 2.

We further quantified the stability of differential expression metrics before and after QC using linear regression (Figure 3a,b). For effect size (log2 FC), the regression equation was y=0.00067+0.999x ($R^{2}=0.992$); for statistical significance (−${log}_{10}$ adjusted *p*-value), the regression equation was y=0.00795+1.0013x ($R^{2}=0.992$). In both cases, the slopes were close to 1.0 and the intercepts approached 0, suggesting that MFF-AE does not introduce systematic bias during outlier removal and preserves the relative magnitude of protein quantification.

We additionally referenced the regression results reported for SEAOP [24] (Figure 3c,d), where the log FC regression was y=−0.21+1.029x and the slope for the ${-log}_{10}$ adjusted *p*-value regression was 1.012. In comparison, the regression parameters of MFF-AE were closer to the identity relationship (slope ≈ 1, intercept ≈ 0), indicating lower perturbation of the differential-expression signal scale. Unlike aggressive filtering methods that might artificially inflate statistical significance, MFF-AE preserves the intrinsic statistical characteristics of the biological data. This confirms that our model effectively removes technical outliers without distorting the underlying biological signals, ensuring the statistical integrity and reproducibility of downstream biomarker discovery.

### 2.5. Robustness to Ultra-Fine-Grained Feature Perturbations

**Figure 4:**
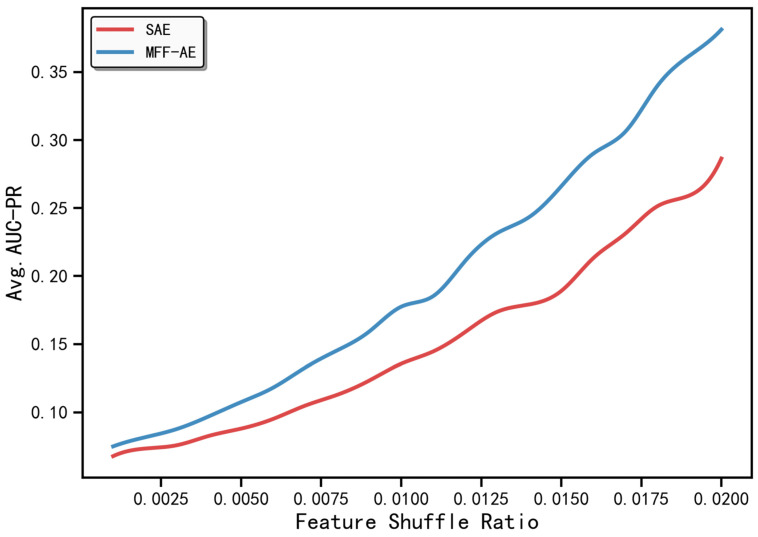
Comparison of AUC-PR performance between SAE and MFF-AE under ultra-fine-grained feature perturbations. The x-axis represents the feature perturbation ratio $r\in \left[0.001,0.020\right]$, and the y-axis shows the mean AUC-PR values across 10 independent trials. MFF-AE consistently outperforms SAE across the entire perturbation range.

The previous experiments evaluated the overall robustness of the model under feature perturbation ratios ranging from 0.01 to 0.40. To further examine the sensitivity of MFF-AE to weak anomaly signals and ultra-fine-grained perturbations, we narrowed the perturbation range to the interval 0.001–0.020, with a refined step size of 0.001. For each perturbation ratio, 10 independent experiments were conducted, and the mean AUC-PR values were computed (Figure 4). In the extremely low perturbation range (r<0.007), both the baseline SAE model and MFF-AE exhibit low AUC-PR values with a slow increase. However, as the perturbation increases (approximately r>0.007), the performance advantage of MFF-AE becomes increasingly evident. Its AUC-PR curve rises more rapidly and clearly surpasses that of SAE. At r=0.020, the AUC-PR of MFF-AE reaches 0.3814, representing an improvement of approximately 33.2% compared with the baseline SAE (AUC-PR = 0.286). These results demonstrate that, relative to the traditional SAE baseline, MFF-AE achieves consistent, stable, and progressively expanding performance gains under ultra-fine-grained perturbations, highlighting its practical value in high-sensitivity anomaly detection tasks.

### 2.6. Model Stability Analysis

To examine the stability of the CLS module and check for potential overfitting, we repeated training with different random seeds and with reduced training-set sizes. Table 2 summarizes the results (mean ± SD over 10 runs). On the full training set, MFF-AE achieved an AUC-ROC of 0.5714 ± 0.0085, with slightly lower variability than the baseline SAE (0.5519 ± 0.0095). The performance remained comparable when the training set was randomly subsampled to 90% and 80%. AUC-PR stayed within 0.101–0.103, and AUC-ROC ranged from 0.5657 to 0.5717, supporting stable behavior across random initializations and moderate reductions in training data.

## 3. Discussion

The goal of MFF-AE is to enable the model to capture global distributional patterns while maintaining high sensitivity to subtle local anomalies. Our approach builds upon an autoencoder backbone, integrates multi-scale feature extraction through a global–local feature fusion module, and introduces a sample identification module to guide the extracted features toward discriminative representations. The superiority of the method has been validated across real-world dataset.

Ablation experiments demonstrate that, compared with the baseline model using only a simple classifier (AE-CLS), the variant incorporating the multi-scale feature fusion module (AE-MSF) improves AUC-PR by 18.7% and AUC-ROC by 9.9%. This effectively addresses the limitations of traditional classifiers in learning intrinsic data representations. Furthermore, adding the CLS module to the AE-MSF variant (yielding the full MFF-AE) further improves performance by an average of 2.7% across metrics. This additional gain stems from the self-supervised regularization signal, which significantly enhances the model’s sensitivity to anomalous samples. The joint optimization of reconstruction and classification losses thus contributes to these overall performance gains. Regarding scalability, although the CLS module introduces a sample-wise classification objective, the computational cost scales linearly with the sample size. For substantially larger datasets, approximation methods such as sampled Softmax or partial instance discrimination can be effectively employed to reduce computational overhead while preserving the regularization effect.

Experimental evaluations on simulated proteomics datasets show that MFF-AE outperforms 15 comparison models for both the AUC-ROC and AUC-PR metrics. In fine-grained perturbation tests, the model demonstrates superior stability and discriminative capability as noise increases. KDE-based visualization indicates that, compared with the MSF model, MFF-AE not only more accurately learns the distribution of normal samples but also induces larger reconstruction errors for anomalous samples. This avoids overfitting to anomalous patterns and provides a more intuitive rationale for its anomaly detection mechanism. Biological validation further shows that differential expression results remain stable after QC; the sets of core DEPs identified before and after outlier removal are largely consistent. Regression analyses reveal high concordance in both *p*-values and log2FC, indicating that removing outlier samples effectively eliminates noise without introducing systematic bias, thereby providing a more reliable data foundation for downstream mechanistic research and biomarker discovery.

## 4. Materials and Methods

### 4.1. Data Sources

To evaluate the effectiveness of MFF-AE, we utilized large-scale datasets derived from label-free quantitative proteomics acquired via data-dependent acquisition mass spectrometry (DDA-MS) and summarized as protein-abundance matrices. The details of the datasets are listed in Table 3.

A unified preprocessing pipeline was applied to both datasets, including global-minimum imputation for missing values and quantile normalization [26]. We selected global-minimum imputation based on an empirical comparison reported in Appendix A (Table A1). Considering that MS-based proteomics data are relatively quantified rather than absolutely quantified, and that unsupervised anomaly detection relies primarily on internal data structure, we adopted a dataset-cleaning paradigm commonly used for batch QC. Specifically, the model was trained on the full sample set, and anomaly scores were computed on the same set using fixed parameters. In the simulation setting, “anomalies” were generated only to provide ground-truth labels for AUC-ROC and AUC-PR evaluation; they were not used for training, hyperparameter tuning, model selection, or thresholding.

For the HeLa-Simulation dataset, we utilized a high-quality HeLa label-free DDA-MS dataset described in our previous work [24]. The initial matrix contains 325 samples (Table 3). After filtering based on protein identification metrics, correlation coefficients, and auxiliary outlier screening, we retained 296 high-quality samples for simulation. The retained samples were randomly divided into 10 groups (~29 samples per group). In each iteration, one group was treated as anomalous and systematic perturbations were introduced via a feature-shuffling procedure. The proportion of replaced feature values was increased from 0% to 40% with a 1% step size to assess robustness across perturbation intensities. Each of the 325 HeLa samples corresponds to an independent DDA-MS QC run and was processed once by the model; no repeated model inference was performed for the same run. Differences in protein identifications across runs are expected for DDA-MS and were partially controlled by the initial filtering; model reproducibility was evaluated by repeating the simulation across perturbation ratios and reporting averaged AUC-ROC and AUC-PR over 10 runs.

To further validate applicability in real-world clinical scenarios, we used a clinically annotated LUAD proteomics cohort from the Chinese Human Proteome Project (CNHPP), originally reported in the integrative LUAD proteomics study. This LUAD dataset consists of 103 paired specimens (103 primary tumor tissues and 103 matched non-cancerous adjacent tissues), totaling 206 samples. Tissue samples were processed using filter-aided sample preparation (FASP) digestion coupled with DDA-MS acquisition, and the raw MS data were processed using MaxQuant (v2.0.3.0) as reported in the original publication [27]. The dataset is publicly available in the iProX repository under accession number IPX0001804000 [28]. To ensure biological relevance, anomaly detection was performed independently within the tumor and normal subgroups. This strategy was employed to distinguish technical anomalies from the intrinsic biological heterogeneity associated with different tissue types.

### 4.2. Data Preprocessing

We performed standardized preprocessing of the raw protein quantification matrices to ensure the consistency and reliability of the model input data. The preprocessing pipeline comprised two key stages: missing-value imputation and normalization.

The raw data generated by MaxQuant contain intensity measurements of thousands of proteins across multiple samples. Due to technical limitations and sample degradation, some missing values were present. To avoid introducing additional noise during imputation, we adopted the global minimum value strategy based on the comparative analysis in Appendix A Table A1.

For each sample with protein measurement, the missing value of a given protein was filled using the global minimum value observed in the entire dataset.

Following imputation, we addressed the wide dynamic range of protein abundances. Physiological protein concentrations differ substantially, often spanning several orders of magnitude. Directly feeding such data into the model would cause the model to be dominated by high-abundance proteins. Therefore, we applied quantile normalization [29]. This method transforms the expression values in each sample to follow a common reference distribution, effectively removing technical variation across samples. The resulting standardized matrix served as the direct input to our MFF-AE model.

### 4.3. Methods

**Figure 5:**
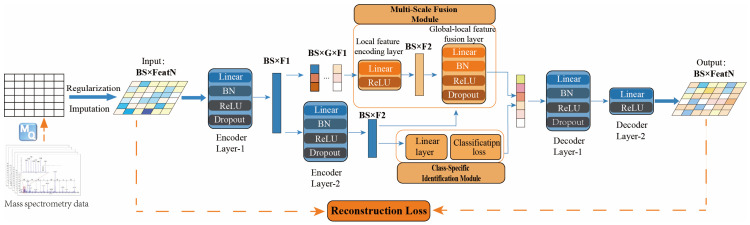
Overview of the MFF-AE model. Raw MS data undergo MaxQuant (MQ) processing, followed by regularization and imputation, to form the input matrix (BS × FeatN). Encoder Layer-1 projects the input into shallow features (BS × F1), which then diverge into two parallel pathways: (I) a Global Pathway (lower branch), where Encoder Layer-2 extracts high-level semantic representations (BS × F2); and (II) a Multi-Scale Feature Fusion Module (upper branch), where features are partitioned into G subgroups (BS × G × F1) to capture local, fine-grained patterns. The global and local features are integrated via a fusion layer and reconstructed by the decoder. A Class-Specific Identification Module applies an auxiliary classification loss on the global representation (Encoder Layer-2 output) to refine discriminability. Solid arrows indicate the main data flow, while dashed arrows represent loss calculations of inputs. Abbreviations: MS, mass spectrometry; BS, Batch Size; FeatN, Feature Dimension (number of proteins); F1/F2, Feature dimensions of intermediate layers; G, Number of feature groups; BN, Batch Normalization; ReLU, Rectified Linear Unit; MQ, MaxQuant.

We present MFF-AE, a Multi-scale Feature Fusion Autoencoder framework for anomaly detection. MFF-AE is a deep anomaly detection model designed to directly ingest MaxQuant protein quantification matrices, eliminating the need for additional data transformation. As illustrated in Figure 5, the MFF-AE architecture comprises three core modules: (1) a Backbone Autoencoder that learns a compressed latent representation of the high-dimensional input and reconstructs it, capturing the essential data structure; (2) a Multi-scale feature fusion module that uses parallel pathways to separately model local details and global context, extract multi-scale features across semantic levels, which are subsequently integrated within a fusion layer. (3) a Class-Specific Identification (CLS) module that leverages the global encoder representation $F_{global}$ (Encoder Layer-2 output) to train a classifier. This module uses self-supervised signals to refine sample-level representations for enhanced discriminability, directly optimizing anomaly detection accuracy.

#### 4.3.1. Backbone Network (AE)

We employ an autoencoder (AE) as the backbone network for efficient data embedding and feature learning. Through an unsupervised reconstruction task, the AE learns to map high-dimensional data into an informative low-dimensional space, capturing its essential characteristics. This forms the foundation for accurate anomaly detection.

The backbone AE comprises an encoder $f_{\theta }(\cdot )$ and a decoder $g_{\phi }(\cdot )$. The encoder consists of several sequentially stacked blocks that progressively transform the input into a low-dimensional latent vector ($z_{i}=f_{\theta }(x_{i})$). Each block contains a linear layer, batch normalization [30], ReLU activation, and dropout [31]. The decoder reconstructs the latent vector back to the original input space. The reconstruction of i-th sample ${\hat{x}}_{i}$ is defined as

(1) $${\hat{x}}_{i}=g_{\phi }(z_{i})=g_{\phi }(f_{\theta }(x_{i})),$$

The model is trained to minimize the reconstruction loss, which is defined as the mean squared error between the input and the reconstructed output:

(2) $$L_{rec}=\frac{1}{N}\sum_{i=1}^{N}{\left\|x_{i}-{\hat{x}}_{i}\right\|}_{2}^{2},$$

where N is the number of samples, $x_{i}$ is the input feature vector of the i-th sample. The low-dimensional representation learned by the backbone network captures the core structure of the data and forms the basis for subsequent anomaly discrimination.

#### 4.3.2. Multi-Scale Feature Extraction and Fusion Module (MSF)

While traditional autoencoders excel at capturing global structure, they often overlook fine-grained patterns embedded within specific feature subspaces [32]. Unlike image data where ‘local’ refers to spatial proximity, in proteomics, technical anomalies typically manifest as subtle shifts within specific subsets of proteins. Focusing solely on the global view may obscure these signals due to the high dimensionality. To better exploit the multi-scale information in proteomic data and capture localized patterns effectively, we introduce a Multi-Scale Feature Fusion (MSF) module between the encoder and decoder.

The MSF module takes the feature tensor $F_{1}=f_{\theta }(x)$ from the first encoder layer as its input. First, we partition $F_{1}$ along the feature dimension into *G* non-overlapping subspaces [33]. This groups proteins into independent feature sets, enabling the extraction of subspace-specific representations ($F_{local}$) that capture localized variations. In parallel, the original feature $F_{1}$ undergoes further processing through the second encoder layer to obtain a global representation ($F_{global}=f_{\theta }(F_{1})$). The local and global features are then concatenated and integrated through a nonlinear fusion layer:

(3) $$F_{fusion}=\sigma (BN(W\left[F_{global}∥F_{local}\right]+b)),$$

where ∥ denotes feature-wise concatenation, W and b are learnable parameters, σ(·) is the ReLU activation function, and $BN\left(\cdot \right)$ represents batch normalization. The fused representation $F_{fusion}$ is passed to the decoder for reconstruction. This design enables the model to jointly preserve both the global structure and the fine-grained subspace details of the MS data.

#### 4.3.3. Class-Specific Identification (CLS) Module

While reconstruction error is effective for identifying many anomalies, we observed that some anomalous samples do not yield significantly higher reconstruction errors. This occurs because an autoencoder can occasionally overfit to certain anomalous patterns during training, enabling it to reconstruct them and thus diminishing its sensitivity.

To address this limitation and enhance the model’s discriminative power, we introduce a Class-Specific Identification (CLS) module as an auxiliary self-supervised objective. This module incorporates a self-supervised signal by attaching a linear classifier on top of the global representation $F_{global}\in R^{B\times F2}$. The classifier produces class probabilities via a Softmax function:

(4) $$P_{class}=Softmax({W_{c}\cdot F}_{global}+b_{c}),$$

where $W_{c}$ and $b_{c}$ are learnable parameters, and c is the identification number of sample. It is trained using the cross-entropy loss:

(5) $$L_{cls}=-\frac{1}{N}\sum_{i=1}^{N}\sum_{j=1}^{C}y_{c}\log\left(P_{c}\right),$$

where N is the total number of samples, and $y_{c}$ is the true label (typically one-hot encoded, meaning $y_{c}=1$ if the sample belongs to class c, and 0 otherwise). Because the CLS targets are instance indices rather than anomalous categories, the classification loss remains well-defined even if a small fraction of anomalous samples is present during training. This auxiliary task encourages the encoder to learn discriminative representations that separate samples, reducing the tendency of the autoencoder to learn a trivial identity mapping.

Although the number of classes equals the number of training samples, the CLS objective is not designed to build an identification system. The classifier is used only during training. It is not used for inference. The risk of learning trivial sample identifiers is constrained by the low-dimensional bottleneck of $F_{global}$ and the joint optimization with the reconstruction objective. This coupling forces the representation to preserve structural information needed for faithful reconstruction. Therefore, CLS acts as a regularizer that encourages a more separable latent space. It also reduces accidental overfitting to anomalous patterns. A related concern is whether instance discrimination may inadvertently encode experimental batch effects. In our pipeline, normalization, regularization and imputation are applied before model training. These steps mitigate systematic batch-related variation. Moreover, CLS is optimized jointly with reconstruction objectives on the bottleneck representation. This discourages learning batch-specific artifacts that do not contribute to faithful reconstruction.

The overall training objective combines both losses:

(6) $$L_{total}=L_{rec}+L_{cls},$$

### 4.4. Model Runtime Environment and Hyperparameters

The experimental environment consisted of a Linux server running Ubuntu 22.04.5 LTS. The system was equipped with dual Intel Xeon Silver 4310 CPUs (2.10 GHz, 48 threads total), 251 GB of RAM, and four NVIDIA GeForce RTX 4090 GPUs, each with 24 GB of VRAM. The driver version was 560.35.05, corresponding to CUDA 12.6. The algorithms were implemented in Python 3.7 using the PyTorch library within the Anaconda3 distribution. Key hyperparameters for model training included: a learning rate of 5 × 10^−4^, a batch size of 16, a dropout rate of 0.2, and the number of feature groups was set to G = 8 based on empirical optimization. It offers sufficient granularity to capture localized anomaly patterns, which are often obscured in global views. Simultaneously, it preserves enough feature density within each subspace to embed proteins.

### 4.5. Evaluation Metrics

To comprehensively assess the anomaly detection performance of the proposed model on proteomics data, we adopted the Area Under the Receiver Operating Characteristic Curve (AUC-ROC) and the Area Under the Precision–Recall Curve (AUC-PR) as the primary evaluation metrics.

AUC-ROC reflects the model’s overall ability to distinguish between normal and anomalous samples; values closer to 1 indicate better overall discrimination performance. It provides a threshold-independent evaluation of a model’s global capability to separate normal and anomalous samples [34]. It is computed based on the true positive rate (TPR) and the false positive rate (FPR):

(7) $$TPR=\frac{TP}{TP+FN},\;\mathrm{FPR}=\frac{FP}{FP+TN},\;AUC-ROC=\int_{0}^{1}TPR(FPR)dFPR,$$

where TP (True Positive) is the number of correctly identified anomalous samples, FN (False Negative) represents is the anomalous samples incorrectly predicted as normal, FP (False Positive) represents normal samples incorrectly predicted as anomalous, and TN (True Negative) denotes the number of correctly identified normal samples.

However, due to the inherently imbalanced nature of proteomics data—where anomalous samples are extremely scarce—AUC-ROC alone may obscure the model’s misclassification of minority-class samples. Therefore, we additionally employ AUC-PR as a complementary metric, which focuses on the precision and recall of the positive class (anomalous samples) and has been shown to more accurately reflect true detection performance in highly imbalanced scenarios [35,36]:

(8) $$Precision=\frac{TP}{TP+FP},\;Recall=\frac{TP}{TP+FN},\;AUC-PR=\int_{0}^{1}Precision(Recall)dRecall$$

By jointly considering both metrics, we obtain a more robust and objective evaluation of model performance from two complementary perspectives: global discriminative ability and effectiveness in capturing rare anomalies.

## 5. Conclusions

In conclusion, we presented MFF-AE, a robust quality control method for proteomics that integrates global and local features to detect subtle anomalies. Our results confirmed that MFF-AE outperforms existing baselines in sensitivity and stability and ensures the statistical integrity of downstream biological analysis. Despite the strong detection performance demonstrated in our experiments, there remains room for future improvement. For example, the current feature partitioning in the MSF module still relies on a manually predefined group number G, and future work may explore adaptive feature grouping mechanisms. Furthermore, while the global minimum imputation strategy proved effective in this study, future work will strictly evaluate the sensitivity of the model to various preprocessing and normalization techniques to ensure broader applicability. Additionally, model generalization under extreme class imbalance requires further enhancement. In subsequent work, we aim to investigate more interpretable feature interaction mechanisms to further improve the applicability and interpretability of the model in complex proteomics datasets.

## Figures and Tables

**Table 1:** Mean and Standard Deviation of AUC-ROC and AUC-PR for Four Model Variants at a Feature Perturbation Ratio of 0.01.

Metrics		SAE	AE-CLS	AE-MSF	MFF-AE
AUC-ROC	Mean	0.580	0.641	0.705	0.721
	SD	0.047	0.035	0.038	0.031
AUC-PR	Mean	0.133	0.141	0.168	0.173
	SD	0.030	0.027	0.024	0.024

**Table 2:** Stability under random initializations and training-set subsampling (mean ± SD over 10 runs).

Model	Training Ratio	AUC-PR	AUC-ROC
	0.8	0.1013 ± 0.0124	0.5657 ± 0.0264
MFF-AE	0.9	0.1022 ± 0.0052	0.5717 ± 0.0206
	1.0 (Full)	0.1033 ± 0.0040	0.5714 ± 0.0085
SAE (Baseline)	1.0 (Full)	0.0949 ± 0.0024	0.5519 ± 0.0095

**Table 3:** Details of datasets used.

Dataset Name	Samples	Protein Counts	Anomaly
HeLa-Simulation	325	6448	29
LUAD	206	11,246	-

## Data Availability

Publicly available datasets were analyzed in this study. This data can be found here: iProX repository under accession number IPX0001804000. The source code and simulated datasets generated during the current study are openly available on GitHub at https://github.com/fanguangkui/MFF-AE (accessed on 9 February 2026).

## Abbreviations

The following abbreviations are used in this manuscript:

MS	Mass Spectrometry
QC	Quality Control
MFF-AE	Multi-Scale Feature Fusion-based Autoencoder
DDA-MS	Data-Dependent Acquisition Mass Spectrometry
AUC-ROC	Area Under the Receiver Operating Characteristic Curve
AUC-PR	Area Under the Precision–Recall Curve
LUAD	Lung Adenocarcinoma
DEP	Differentially Expressed Protein
KDE	Kernel Density Estimation

## Appendix A

### Comparison of Missing Value Imputation Strategies

To validate the choice of the global minimum imputation strategy, we compared its performance against Mean Imputation, Linear Interpolation, and KNN Imputation (k = 5) using the Isolation Forest algorithm. The Score Difference represents the margin between the average anomaly score of outliers and normal samples.

**Table A1:** Performance comparison of different imputation strategies.

Imputation Method	Fake Detection Ratio	Score Difference
Global Minimum (Proposed)	0.1	0.0153 (Highest)
Linear	0.1	0.014286
KNN	0.1	0.012587
Mean	0.1	0.012391
